# Efficacy of Occupational Performance Coaching with and without Four Quadrant Model of Facilitated Learning for mothers of children with specific learning disorder: Study protocol for a randomized controlled trial

**DOI:** 10.1016/j.conctc.2022.101009

**Published:** 2022-09-22

**Authors:** Amin Ghaffari, Akram Azad, Mehdi Alizadeh Zarei, Mehdi Rassafiani, Hamid Sharif Nia

**Affiliations:** a Rehabilitation Research Center, Department of Occupational Therapy, School of Rehabilitation Sciences, Iran University of Medical Sciences (IUMS), Tehran, Iran; b School of Allied Health, Exercise and Sports Sciences, Charles Sturt University, Albury, Australia; c Department of Nursing, Amol Faculty of Nursing and Midwifery, Mazandaran University of Medical Sciences (MUMS), Sari, Iran

## Abstract

**Background:**

In addition to academic problems, children with a speciﬁc learning disability (SLD) encounter problems with participation in activities outside school.

**Purpose:**

To investigate the efficacy of Occupational Performance Coaching (OPC) with and without Four Quadrant Model of Facilitated Learning (4QM) in the mothers of children with SLD.

**Method:**

A single-blinded, parallel-group randomized clinical trial will be carried out. Mothers of children with SLD will constitute the participants and be allocated to experimental (OPC+4QM) and control (OPC alone) groups.

**Key issues:**

Children's occupational performance and satisfaction, participation in activities outside school, goals attainment, executive function, and academic achievement along with self-efficacy of mothers will be measured in baseline, post-intervention, and one-month follow-up stages.

**Implications:**

An OPC intervention protocol may improve children's participation in activities outside school and may help clarify whether 4QM promotes maternal empowerment and better results for children with SLD.

**Trial registration:**

Clinical Trials, IRCT20140416017301N9. (https://en.irct.ir/trial/55842)

## Abbreviations

SLD: Speciﬁc Learning Disability
OPC: Occupational Performance Coaching
4QM: Four Quadrant Model of facilitated learning

## 1 Introduction

Specific Learning Disorders (SLD) is a neurodevelopmental disorder characterized with declined academic capability in the early years of elementary school and is two to three times more common in boys than in girls [1]. Children with SLD have a normal intelligence (IQ > 70); however, experience difficulties in reading, writing, speaking, or executive functions [2]. The prevalence of SLD among school-age children is reported 4.58–7% in Iran [3,4] and 5–15% in other countries [1].

Based on the DSM-V (Diagnostic and Statistical Manual of Mental Disorders), the severity of SLD is measured by the extent of participation in occupational performance areas, especially activities outside of school [1,5]. Activities outside school include basic activities daily of living (BADL), instrumental activities daily of living (IADL), play, leisure, social participation, education (except academic functions), work, and sleep/rest [5,6].

Studies show that the neuro-motor developmental deficits in children with SLD have caused limitations in activities outside school such as self-care activities (e.g. shoe tying, buttoning up clothes and getting dressed), productivity activities (e.g. legible writing and doing homework), and leisure activities (e.g. playing with peers and painting) [[7], [8], [9], [10]]. Furthermore, children with SLD often show failure in executive function skills and thus struggle with acquiring new skills in almost every domain of life [[11], [12], [13]]. The extra effort for learning new tasks, such as tying shoe laces or riding a bike, results in avoiding these activities and leads to participation restrictions in activities outside school [8,14]. Unfortunately, only the academic issues have received the attention while there is a significant correlation between academic skills and participation in daily life activities in children with SLD [15]. Based on the Occupational Therapy Practice Framework (OTPF-4th edition), “participation in occupations is a key part of development and life experience for man, which provides the opportunity to gain competencies and skills and find meaning and goal in life” [16]. Therefore, participation in activities outside school is an important indicator of health and well-being that helps to encourage positive development into adulthood and should be considered as a major goal of rehabilitation services [17,18].

Studies have reported defects in executive function domains such as verbal working memory, sequencing [19,20], set-shifting [21], planning, timing [22], and response inhibition [23] in children with SLD. Executive function skills shape various routine and non-routine activities of daily life and also participation in the various areas of occupation [[24], [25], [26]]. Children suffering from executive dysfunction may have deficits in dealing with complicated occupations with dynamic task demands and multiple steps, like IADL and social participation which can lead to restrictions in their life roles [27,28]. Performing unprecedented tasks also creates a challenge for these children as they cannot perform actions they have learned to perform automatically (inhibitory control and cognitive flexibility) for fulfilling new challenging activities in different contexts (transfer and generalization). Therefore, these children are limited in terms of participation in everyday activities [29,30].

The ICF-CY (International Classification of Functioning, Disability, and Health-Child and Youth) as a biopsychosocial framework, indicates that child's performance, health, and wellbeing depend on the dynamic interaction between the activities, structures and body functions, and participation taking into account the personal and the environmental factors [31]. Therefore, facilitating children's participation in activities outside school, in addition to improving body function and structure such as executive functions increase their independence in occupational performance.

Mothers with SLD children spend long hours and a great deal of energy on the education of these children compared to their siblings [32]. Sahu et al. (2018) showed in their exploratory study on the perception of families of SLD children that different supportive strategies are needed to empower mothers of these children [33]. In addition, such services warrant mothers' training to support the learning skills of their child [34]. On the other hand, studies on children with special needs show that the most effective services emphasize the family's role in treatment [35,36]. Therefore, empowering mothers to identify barriers and facilitators of their child's participation in activities outside school and solving their problems through collaboration with a therapist is one of the main priorities of children's rehabilitation, which in turn can strengthen their self-efficacy.

Occupational Performance Coaching (OPC) is a family-centered, occupation-based, solution-focused intervention that directly targets children's participation in activities outside school through working with their mothers as the mediators of change [37,38]. The OPC incorporates three key domains including information exchange, emotional support, and a structured process that mixes performance analysis frameworks of current performance with coaching techniques to involve mothers in goal-specific and collaborative processes. Goals are set through exploring available options in the child (knowledge, motivation, and ability), task (sequence, steps, and standard expected), and environment (physical and social) using collaborative performance analysis (CPA). To explore options includes leading the mother to examine her child's current level of performance, determine the ideal level of performance they would like to happen to her child, and identify bridges, and barriers to the child's success in performance. Eventually, planning and taking action to attain occupational performance goals related to child participation happen in the home and community [38]. Through OPC, mothers learn to develop their problem-solving ability by identifying novel, ambitious, but highly individualized, and directly applied strategies to improve their child's participation in activities outside school [39].

As mentioned, children with SLD have difficulty acquiring new skills of daily life activities [8,9]. Skill learning is a major step in achieving occupational goals that is attained through utilizing learning and teaching as major ways of intervening pediatric practice [40]. According to Vygotsky's theory, children need support to learn the necessary skills and develop their abilities. In other words, progress toward independence and autonomy in performing tasks is made possible through systematic facilitator support [41]. This concept is consistent with the Mosston and Ashworth's study that stated teaching a task is like a spectrum that starts with command-style (teaching key elements directly to the learner along with the way of reacting to task demands) and ends with self-teaching (motivates learners to take into account the challenges to perform the activity and the way of achieving independence and autonomy through decision-making processes) [42]. Thus, selecting effective and systematic teaching-learning strategies based on the body functions of SLD children (e.g. executive functioning), mothers' awareness of learning level, and changing needs of children during the acquisition of new skills seems to help to booster OPC and finally better improve participation in children's activities outside school.

One of the models of learning facilitation in the teaching-learning approach is the *Four Quadrant Model of Facilitated Learning* (4QM), which was first proposed by Gerber in 2007 [43]. The 4QM provides the various systematic physical and cognitive learning strategies needed in guiding children to carry out occupational tasks autonomously and independently [44]. Executive function skills such as initiation, termination, planning, organization, self-monitoring, self-control, working memory, time management, and organization are included in the four quadrants of this model [45]. In quadrant 1, learning strategies are direct, initiated by the mother, and determine the features of the task and/or the performance requirements. The rest of indirect strategies, which are mother-initiated, are in quadrant 2. Such strategies encourage decision-making by the child. Quadrant 3 encourages the child through the use of overt self-prompts to recall key points and finally, quadrant 4 includes a range of self-regulatory metacognitive and cognitive strategies that help the child's autonomy and independence [44]. It is necessary to mention that, the 4QM can be employed only when the children with SLD are judged to have the necessary performance components to complete the activity [43]. In other words, after identifying barriers to achieving the goal (barriers related to the child, activity, and context) through OPC, independence in the goals of participation seems to be facilitated with 4QM.

The OPC is potentially effective through empowering mothers to improve children's participation in various life situations [37,38,46]. On the other hand, adding 4QM as a reinforcement tool to OPC, increases the learning needs of mothers and facilitates teaching necessary skills to children with SLD. The most relevant evidence to this hypothesis is the recent single-subject study of the effectiveness of OPC with 4QM in the occupational performance of six children with SLD [47]. As far as the authors know, there has been no study on rehabilitation interventions to improve participation in children with SLD. Studies are limited to participation patterns and environmental factors [2]. Generally, participation of children with SLD has not received the attention it merits. Thus, the present study explores the use of 4QM, in addition to OPC, in the experimental group and investigates the effect of this intervention on improving the outcomes for children with SLD.

The primary objectives are to examine the efficacy of the OPC approach with and without 4QM on occupational performance, satisfaction, and achievement of goals (transfer and trained goals) and participation in activities outside school for SLD children. In addition, the efficacy of OPC+4QM versus OPC on similar results will be examined. The second-level goals are to examine and compare OPC+4QM and OPC efficacy on executive performance (such as organization, cognitive flexibility, and mental planning); to investigate and compare academic achievement; and to examine and compare self-efficacy in mothers of children with SLD. The trial's hypotheses state that OPC+4QM and OPC are effective in improving outcomes in children and parents and the experimental group submitted to OPC+4QM have better effects on the main and secondary outcomes immediately and one month after the interventions.

## 2 Method

### 2.1 Design and setting

The study will be a single-blinded, parallel-group, and randomized clinical trial with an add-on component. The OPC model will be implemented for all participating mothers. Four extra training sessions of 4QM will be provided to mothers in the experimental group. The Ethics Committee of the Iran University of Medical Sciences, Tehran, Iran (IR.IUMS.REC.1400.060) approved the study. It was also registered as a randomized controlled trial (IRCT20140416017301N9) at http://www.irct.ir/.

The study place will be the centers for special learning problems affiliated with Exceptional Education in Tehran. The first author (A.GH) will be responsible for the study processes. He is a clinician with experience and an occupational therapist and has experience working with mothers on OPC and 4QM interventions.

### 2.2 Participants and procedures

The mothers with DSM-5 diagnostic criteria for SLD will be selected. Participants will be selected through active search in four special learning problems clinics affiliated with Exceptional Education in Tehran, Iran. The children with SLD are those referred to the clinics by public schools.

In the case of children, the inclusion criteria are: (a) age between 7 and 12 years; (b) diagnosed SLD according to the Diagnostic and Statistical Manual of Mental Disorders (5th ed.; DSM–5); (c) not having vision and hearing problems; (d) no comorbid psychiatric disorder according to the Persian version of Child Symptom Inventory-4 (CSI-4); and (e) Intelligence quotient higher than 70 (based on the Persian version of the Wechsler Intelligence Scale for Children-4th Edition (WISC-IV). Cases with significant symptoms according to CSI-4 will be referred to a psychiatrist.

In the case of mothers, the inclusion criteria will be age range of 25–50 years and a high school diploma at least. Mothers who are in charge of caring for another person with disabilities, or severely depressed (Depression, Anxiety, and Stress Scale-21 [DASS-21]) will be excluded [48].

**Fig. 1:**
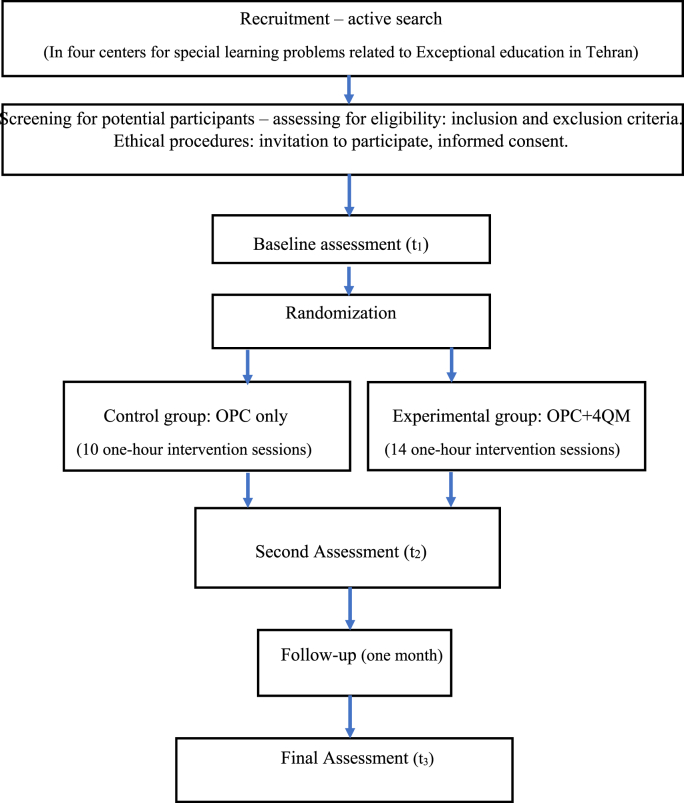
Flowchart of the study.

To assess eligibility criteria, mothers of children with SLD will be interviewed and advised regarding the type of study. Selected mothers will need to sign an informed letter of consent. An external examiner with training (an occupational therapist) will examine the children and their mothers using CSI-4 and DASS-21 respectively. In addition, a psychologist will examine children using WISC-IV (Fig. 1).

### 2.3 Participant timeline

**Fig. 2:**
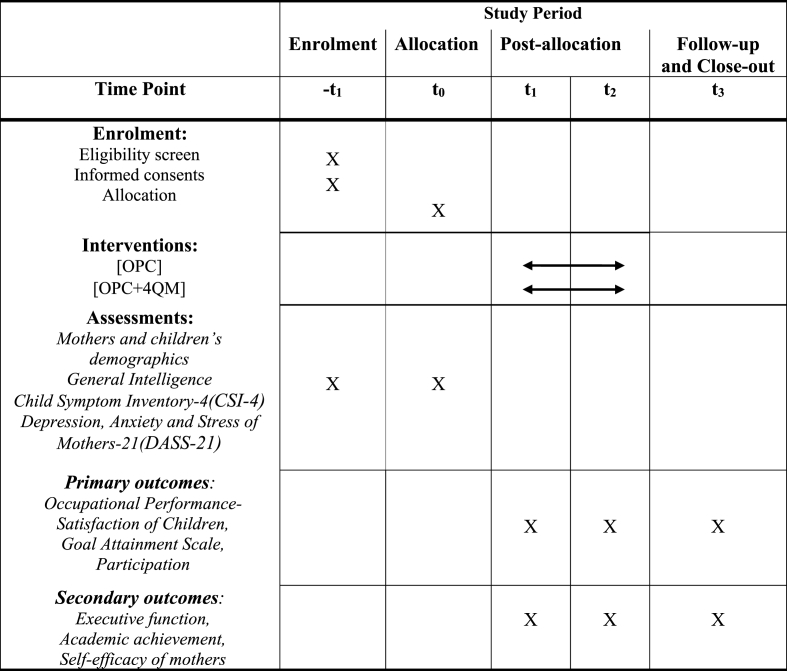
Schedule of enrolment, interventions, and assessments.

Participant timelines initiate eligibility screening through allocation, pre/post-intervention, intervention, and follow-up (Fig. 2).

### 2.4 Sample size

Sample size calculations were performed in G-Power software based on the COPM test as the main variable of the study. A two points change in this variable between the intervention and control group will be considered as significant difference [49]. Considering α-value of 5%, the power of 95%, the standard Cohen's effect size of 1, the required sample size was obtained equal to 27 for each group, and assuming 10% dropout rate, 30 subjects were selected for each group. Furthermore, since the sample size was calculated based on the primary outcome, the study is anticipated under-powered for addressing secondary outcomes.

### 2.5 Allocation

Mothers of children with SLD with the inclusion criteria will be assigned with a number. After baseline assessment, the research coordinator will allocate them sequentially (allocation ratio 1:1) to the control or experimental group, based on the random numbers generated by *randomization. com*.

### 2.6 Blinding

In this study, two occupational therapists will support the experimental and control groups. Therefore, given the nature of the therapeutic intervention, the participant (mother) cannot be blinded about their grouping. To decrease participants’ bias, the study hypotheses will not be communicated. Examiner will be kept blinded about allocation of the participants and the order of the assessment. Additionally, data input will be done by a research assistant without a direct role in the intervention. Group allocations will not be revealed to data analysts.

## 3 Interventions

First, necessary explanations about the importance of OPC or OPC+4QM for participation in outside school activities in SLD children will be given to the mothers. To ensure adherence and compliance to treatment, an evaluation form to assess mothers' ability to implement the instruction of each session and important issues during the intervention course will be completed by each participant. Mothers will not be required to quit other interventions, if any, such as psychology or their child's school tutorials; however, they need to avoid engaging in other occupational therapy interventions.

Second, the original OPC (Graham & Rodger, 2010) and 4QM (Greber, 2007) protocols will be used in the study groups [39,50]. Two occupational therapists who have more than 5 years of experience working with mothers with children with neurodevelopmental disorders will conduct the interventions, and both OPC and 4QM protocols are available through manuals. A rater will be present during the intervention sessions in both experimental (OPC+4QM) and control (OPC) groups and complete the occupational performance fidelity measure (OPC-FM) to observe the principles of OPC by the therapist and also to check the quality of behavior of the mothers [[51], [52]]. OPC-FM consists of 18 items, and a higher score indicates a better OPC behavior. The average score will be 34.1/45 for the first session and 41.3/54 for the other sessions. The minimum requirement recommended in the coaching manual will be 80% fidelity in every session [51].

Third, all the participating mothers will be provided with a booklet for the study based on prior experiences containing information related to OPC and an additional part about implementing the 4QM approach and guided discovery to help the improvement of participation in activities outside school-related to their children.

### 3.1 OPC approach protocol

OPC interventions will be delivered for ten 60-min sessions provided once a week. The follow-up period will be one month.

In the first session, after introduction and explanation about the details of the OPC interventions, five goals related to the child's participation in activities outside school (three trained and two transfer goals) will be set by each mother for her child. In the second session, the problem-solving process will begin for the first goal and the printout of the problem-solving process will be given to the mothers. In this session, the mother will be asked to carry out two different actions at least about the desired goal during the next session. In the third session, the first goal will be reviewed and the work on the second and third goals (one or two for each goal) will begin. In the fourth to eighth sessions, progress will be reviewed across the three goals. In the ninth session, the selected goals will be reviewed and the necessary points for the end of the intervention sessions will be discussed. In the tenth session, which is the last session and the end of OPC intervention, a decision is made about the goals that were not achieved. Transfer goals (fourth and fifth goals) will also be implemented by mothers during the follow-up period.

The content of the sessions is based on enabling principles that include three domains: emotional support, information exchange, and structure of the process. The emotional support domain is focused on listening to parents' information and interpretation by the child. The fundamental facilitating actions in this domain are motivators for change, learning needs in implementing change, previous success in enabling performance, expressing empathy, assisting parents in reframing their perceptions of the child, enabling performance and guiding parents’ reflections and choices of action, encouraging persistence, and future independent problem-solving. In the information exchange domain, a discussion takes place between the mothers and the therapist regarding (a) collaborative performance analysis, (b) understanding typical development, (c) impairments and related challenges in children with SLD, (d) teaching and learning strategies, (e) finding and accessing community resources, and (f) implementation of guided discovery in different settings. In the structured problem-solving process, priority goals related to activities outside school that are chosen by the mother and indicate worries about her child will be discussed by the therapist and the mother and explore options will be examined. Then, the actions are planned and the results will be checked [39,53,54] (Table 1).

**Table 1:** Coaching protocol based on OPC principles.

OPC domains	Key facilitating actions
Emotional Support	• Listen
	• Emphasize
	• Reframe
	• Guide
	• Encourage
Information Exchange	• Collaborative performance analysis
	• Typical development
	• Health conditions and impairments
	• Teaching and learning strategies
	• Specialized Strategies
	• Community resources and Entitlements
Structure the Process	• Set Goals
	• Explore Options
	• Plan Actions
	• Carry out Plan
	• Check Performance
	• Generalize

### 3.2 4QM protocol

The OPC+4QM interventions will be provided once a week with fourteen 60-min sessions (ten sessions for OPC + four sessions for 4QM). The follow-up period will be one month. Four sessions of 4QM will be scheduled in four quadrants during the OPC sessions to achieve the identified goals. After analyzing each goal in the OPC approach by using collaborative performance analysis, 4QM will be employed for the plan action of that goal. Also, the principles of teaching and learning will be present both in OPC and 4QM sessions, but in Four sessions of the 4QM, various cognitive and physical learning strategies are organized and implemented in the form of 4 quadrants.

The first 4QM intervention sessions along with the first OPC session introduce the 4QM, its components, and presents the printed forms of 4QM for each goal to the mothers. The second, third, and fourth sessions of 4QM interventions will be done along with the OPC sessions related to the first to third selected goals to train and implement the goals in the format of 4QM.

Integration of two continua is used in 4QM given the (i) directness of the strategy (first direct and then indirect) and (ii) clustering the initiation source (first the mother as a facilitator and then the child as a learner) to prompt teaching-learning strategies given the needs of the child. In the quadrant 1, the characteristics and requirements of the task are determined. Strategies including demonstration, explicit instruction/explanation, lower-order questions, and physical patterning, will be initiated by the mother (facilitator) directly. In the quadrant 2, the child will be supported in carrying out tasks through involving them in decision-making processes. Indirect strategies including feedback, higher-order questions, nonverbal prompts, physical prompts, and think-aloud modeling will be initiated by the mother (facilitator). The quadrant 3 will focus on identifying the steps of the activity and child (learner) driven processes and highlights direct strategies like mnemonics, priming, visual cues, verbal self-instruction, and kinesthetic self-prompting that a child (learner) might overtly use to improve their performance through concentrating on key points. Quadrant 4 indicates autonomous performance by the child (learner) through indirect strategies like self-instruction, self-questioning, mental imagery, problem-solving, self-monitoring, and automaticity [44,55,56] (Table 2).

**Table 2:** The four-quadrant model of facilitated learning in the current study.

Quadrants	Key Concept	Strategies
Quadrant1 (Direct, Mother-Initiated)	Task Specification	• Explicit instruction/explanation
		• Demonstration
		• Physical patterning
		• Lower order questions
Quadrant 2 (Indirect, Mother-Initiated)	Decision-making	• Higher order questions
		• Feedback
		• Physical prompts
		• Nonverbal prompts
		• Think-aloud modelling
Quadrant 3 (Direct, Child-Initiated)	Key points	• Priming
		• Mnemonics
		• Verbal self-instruction
		• Visual cues
		• Kinesthetic self-prompting
Quadrant 4 (Indirect, Child-Initiated)	Autonomy	• Mental imagery
		• Self-instruction
		• Self-questioning
		• Self-monitoring
		• Problem solving
		• Automaticity

### 3.3 Goal setting

Mothers need to speak about their child's activities outside school and to set three objectives for the intervention and two extra goals (transfer goals) as the measure of outcome for the transfer [41]. The occupational therapist will ask mothers to assign a point to each goal with the 10-point scale scoring system of the COPM (Canadian Occupational Performance Measure). To make mothers more familiar with the details of activities outside school, during the semi-structured interview COPM, CPAS-P (Children Participation Assessment Scale -Parent Version), including 71 items of different areas of children's occupational performance will be provided to them. In addition, simultaneous with the COPM, the Goal Attainment Scale (GAS) will be used to examine the achievement of the desired goals.

## 4 Data collection

### 4.1 Characteristics of participants

Demographic information of mothers such as age, job, and level of education and demographic information of children such as age, school grade, and gender will be collected.

The WISC-IV (Persian version of the Wechsler Intelligence Scale for Children-Fourth Edition) will be used to test the intelligence of the children with SLD in this study. Perceptual reasoning, Verbal comprehension, processing speed, working memory, and full intelligence scales are evaluated by the test. The validity and reliability of the tool for Iranian children in the age range 6–16 years have been confirmed [57]. Furthermore, the WISC-IV scales and cognitive assessment system (CAS) test are acceptably correlated for children suffering learning disorders. There is also concurrent validity between the two tests and the scales [58].

The Child Symptom Inventory-4 (CSI-4) will be used to screen children aged 5–12 years for eighteen behavioral and emotional symptoms and measure comorbidity. This 4-point Likert scale is based on the DSM-V and demonstrates the frequency of psychiatric symptoms seen in the children [59]. The questionnaire has two (parent and teacher) versions. The parent version will be used in this study. Test-retest reliability of the Persian version of CSI-4 (parent version) for eleven disorders is 0.29–0.76 [60].

The DASS-21 (Depression, Anxiety, and Stress Scale- 21 Items) will be utilized to screen the mental health of participating mothers. The DASS-21 contains 21 items with three dimensions (anxiety, depression, and stress) [61]. The DASS-21 has a desirable test-retest reliability (0.740–0.881) and validity (Cronbach's alpha of stress = 0.67, anxiety = 0.49, and depression = 0.70) was reported for the Persian version of DASS-21 [62].

### 4.2 Outcome measures

The results will be examined before, immediately after, and one month of the intervention. Moreover, the psychometric properties of outcome assessment tools will be checked by professionals previously trained. Assessors (occupational therapists) will be not aware of group allocation and only will be aware of the allocation as the main investigator.

#### 4.2.1 Measures of the primary outcome—the first and second study goals

##### 4.2.1.1 Occupational performance and satisfaction

The COPM (Canadian Occupational Performance Measure) will be utilized for scoring occupational performance and satisfaction by the mothers of children with SLD. As a semi-structured interview, COPM helps mothers identify their goals. The participant is asked to score performance and satisfaction with selected goals related to activities outside school on a 10-point score (ranging from 1 = not satisfied to 10 = satisfied) [63]. Two-point change or more overtime are considered clinically relevant. Test-retest reliability for COPM is measured for various populations and it varies 0.84 to 0.92 [64]. The content validity of the Persian version of the COPM is (80.95 ± 0.222) [65].

##### 4.2.1.2 Achievement of objectives

Goal Attainment Scale will be used to quantify progress towards selected goals related to activities outside school by the mothers of children with SLD. This scale is useful in the family-centered intervention where parents, as ‘experts’, can make important contributions to their children's success [66]. The GAS includes five scores (−2 to +2). A score of zero means the expected level of performance after some time, scores of −2 and −1 indicate reaching a level lower than expected, and scores of +2 and + 1 indicate reaching a level higher than expected [67]. The inter-rater reliability of the scale is 0.67 in different populations and content validity (r = 0.86) has also been reported for pediatric disorders [68].

##### 4.2.1.3 Participation in activities outside of school for children

The CPAS-P (Children Participation Assessment Scale in Activities Outside of School-Parent Version) will be used to assess participation by children outside school in two key life situations (community and home) [5]. This tool is designed for children in the 6–12 years age range. The tool contains 71 items categorized in eight areas of occupations including BADL, IADL, leisure, play, work, education, social participation, and sleep/rest. The parents should report the diversity of activities for each activity that the child performs [6]. Intraclass correlation coefficient (ICC) and Cronbach's alpha for the Persian version of CPAS-P were 0.91–0.94 and 0.90 to 0.95, respectively [69].

##### 4.2.1.4 Transfer of skills

The transfer goal (fourth and fifth goals) will not be covered in the intervention. The occupational satisfaction and performance in the children on the transfer goal will be measured using COPM's scoring system. Furthermore, achievement of these objectives will be measured using GAS at baseline, post-intervention, and after a 1-month follow-up. Capistran and Martini reported the use of extra goals to evaluate transfer with children [70].

#### 4.2.2 Secondary outcome measures—the third and fourth study objectives

##### 4.2.2.1 Executive function skills for children

The BRIEF (Behavior Rating Inventory of Executive Function)-Parent Form will be utilized to determine the outcome. The mothers will rate their children's behavior and metacognitive function in the natural context (home or school). This questionnaire is designed for children in the 5–18 years age range and consists of 86 items categorized into eight separate subscales (Working Memory, Initiate, Organization of Materials, Plan/Organize, Inhibit, Monitor, Shift, and Emotional Control). The total score of these subscales is called the Global Executive Composite (GEC) that is divided into two broad scales of Behavior Regulation Index (BRI) and Metacognition Index (MCI) for scoring [71]. The GEC, BRI, and MCI scale test-retest coefficients were 0.80, 0.81, and 0.83, respectively [72]. As to the internal consistency of the BRIEF (parent-form) Persian version, Cronbach's alpha for GEC was 0.86 [73].

##### 4.2.2.2 Academic achievement of children

Grade Point Average (GPA) will be used to measure academic achievement of children in this study. This score is the average point in the three subjects of mathematics, reading, and spelling; which are three courses in which children with SLD are more likely to have academic failure [15]. Mothers are required to receive their child's GPA from the teacher of the school before, after, and one month after the intervention.

##### 4.2.2.3 Self-efficacy of mothers

The Sherer General Self-Efficacy Scale (SGSES) will be used to measure the self-efficacy of mothers of children with SLD. It features a five-point rating scale with 17 items, so that the higher the score the better the self-efficacy. The internal consistency and test-retest reliability of this scale are 0.86 and 0.76 respectively [74]. Confirmation of several predicted conceptual relationships between the Sherer General Self efficacy subscales with personal characteristics like self-esteem provided evidence of construct validity and internal-external control [75].

## 5 Data management

An assistant will enter the data into SPSS-Vesrion22®. The participants' data will be stored on a password-protected lab computer. According to the Research Ethics Committee, all data including evaluation forms, intervention checklists will be kept in in the laboratory for five years.

### 5.1 Adverse events

During the course of assessment or intervention, we will record all adverse events like pain, discomfort, and disagreement. In the case that the adverse events are related to the study plans, modification or termination of activities or sessions can be considered. The mothers will undergo a medical check to examine any significant or unexpected side effect.

### 5.2 Data analysis

To analyze the first and second objectives, (a) mean and median changes in the satisfaction and occupational performance scores of the first three goals chosen by the mothers from the COPM from baseline (t_1_) to post-intervention (t_2_) and a 1-month follow-up (t_3_); (b) mean and median changes in the scores of the diversity of participation in activities outside school provided by CPAS-P from t_1_ to t_2_ and t_3_; (c) mean and median changes in goal attainment in the scores of the first three goals chosen by the mothers on GAS from t_1_ to t_2_ and t_3_; and (d) mean and median changes on occupational performance and satisfaction and goal attainment in the scores on COPM and GAS on transfer goals (The fourth and fifth goals chosen by the mothers) to analyze the transfer of skills from t_1_ to t_2_ and t_3_ will be used.

To analyze the third and fourth objectives (a) mean and median changes in the total score of executive function on the BRIEF from t_1_ to t_2_ and t_3_; (b) mean and median variations in educational status on the transcript from t_1_ to t_2_ and t_3_; and (c) mean and median variations in the self-efficacy of mothers on the SGSES from t_1_ to t_2_ and t_3_ will be used.

### 5.3 Statistical methods

Qualitative and quantitative variables are explained using relative and absolute frequencies, as well as central tendency, variance, and position measurements. To examine the homogeneity in characterization variables (age, gender, child's level of education, parent's level of education-possible confounding variables) chi-square test for qualitative variables and *t*-test for quantitative variables will be used. The Shapiro-Wilk test will be used to assess the normality of variables. Analysis of variance (ANOVA) will be utilized for comparison of the efficacy of OPC+4QM versus OPC on the primary and secondary results. The multivariate models include time and group interactions and Intention to Treat (ITT) will be used for participants who do not complete the interventions; pre-test score will be used instead of the post-test score [76]. A 2 tailed p < 0.5 will be considered significant and effect size (partial η^2^) will be calculated using R software-version 3.5.0.

## 6 Implications

It is critical to measure mother's contribution to occupation performance if any and to determine the participation of their SLD children using the OPC. In addition, we need to examine any difference in the case of providing support to mothers using a learning model based on 4QM. Moreover, it is anticipated that the study will yield a great deal of data about mechanisms of intervention effectiveness, family needs, and the cost-effectiveness of an intervention of this nature. In conclusion, future studies using a randomized controlled design and representative and large sample group would be appropriate to test outcomes over long term in mothers of children with SLD.

## Key messages

- Participation in activities outside school is an important aspect of the occupational performance of children with SLD that should be studied.
- The OPC as a family-centered, occupation-based, and solution-focused intervention may improve participation in activities outside school for children with SLD.
- Adding 4QM to OPC may enhance the learning needs of mothers from their child's level of learning, which leads to superior results in outcomes for mothers and their children with SLD.

## Availability of data and materials

The datasets and informed consent materials used and/or analyzed during the study will be available from the corresponding author upon request.

## Dissemination policy

The findings will be published in peer-reviewed journals. Communications will be submitted to conferences and a brief video clip will be developed and made available on the internet to foster knowledge translation.

## Declaration of competing interest

The authors declare that they have no known competing financial interests or personal relationships that could have appeared to influence the work reported in this paper.
